# Assessment of the Quality Attributes of Oat β-glucan Fortified Reduced-Fat Goat Milk Yogurt Supported by Microfluidization

**DOI:** 10.3390/foods12183457

**Published:** 2023-09-16

**Authors:** Elif Ayse Anli, Asuman Gursel, Ayse Gursoy, Behic Mert

**Affiliations:** 1 Department of Dairy Technology, Agricultural Faculty, Ankara University, 06110 Ankara, Turkey; gursel@agri.ankara.edu.tr (A.G.); gursoy@agri.ankara.edu.tr (A.G.); 2 Department of Food Engineering, Middle East Technical University, 06531 Ankara, Turkey; bmert@metu.edu.tr

**Keywords:** oat beta-glucan, dietary fiber, goat milk yogurt, high-pressure homogenization, phase separation, thermodynamic incompatibility

## Abstract

In this study, goat milk blends (1.5% fat) fortified with 0%, 0.25%, and 0.50% oat β-glucan were coded as YC, Y1, and Y2 and MFYC, MFY1, and MFY2. Microfluidization was applied at 103.4 MPa pressure in a 100 µm-process chamber at one stage for MFYC, MFY1, and MFY2 prior to yogurt making. Phase separation occurred due to the casein-β-glucan interaction observed at the oat β-glucan ratio (≥0.25%) but was more distinct at 0.50%. Microfluidization solved the textural instability at all ratios of β-glucan; a creamy and less cohesive structure was maintained in all yogurt samples. Among the samples, Y2 and MFY2 were the least viscous (*p* < 0.05), and syneresis was the highest and the lowest for Y2 and MFY1, respectively (*p* < 0.01). Lightness (L*) decreased, and yellowness (b*) and greenness (a*) increased with oat β-glucan concentration (*p* < 0.01) and MFYC. MFY1 and MFY2 were brighter and less green (*p* < 0.05). Microfluidization enhanced sensory attributes and oat β-glucan suppressed the goaty and salty taste, but the cereal taste became more obvious with the increase in the oat β-glucan ratio. Y1 and MFY1 were generally acceptable, and Y2 was less (*p* < 0.01). A liquid-like structure was observed in Y2 and this affected the sensorial perception in Y2.

## 1. Introduction

Goat milk is popular due to its dietetic superiorities, especially for children, older people, and lactating women having digestive difficulties mainly associated with α-casein and β-lactoglobulin protein fractions [1,2]. Goat milk contains fewer allergenic proteins (αs1-casein) and fat globules with smaller diameters that help in terms of easy digestion. Milk proteins with different qualities and at different quantities result in different physicochemical properties in goat milk than in cow milk [1,2,3]. Textural defects, such as a weak texture, a loose gel structure [4,5,6,7], and low viscosity and consistency [8,9], are encountered in yogurt [10,11] due to low or deficient αs1-casein levels [1]. Sensorially, goat milk has a unique goaty and salty taste/flavor [12]. The goaty flavor is related to the octanoic acid present in goat milk in higher amounts and generally diminishes its acceptance by consumers.

Fermented dairy products are fortified with dietary fibers for their physiological functionality, prebiotic effect, structural development, and improvement of sensory properties [13,14,15,16,17]. In goat milk yogurt, textural improvement and masking the goaty and salty taste/flavor through fortification are the first possible applications employed for quality enhancement. β-glucans are water-soluble fibers in cereals with many physiological benefits, such as reductions in blood lipid and blood sugar levels, lowering cholesterol, delaying gastric emptying, supplying immune enhancement, and acting on therapeutic effects for coronary heart diseases [13,14,18,19,20]. According to the American Food and Drug Administration (FDA), the positive effects of β-glucan on health can occur when 0.75 g is consumed per serving [21] and the daily intake of β-glucan recommended by the FDA is 3 g [19,22]. Technologically, β-glucans are also functional depending on their molecular weight (Mw), source of origin, extraction method, and degree of purification [23,24]. Oats are the most important known sources of natural β-glucan; with a content of 3–7% [19,25,26]. Oat β-glucan is a chemically linear polysaccharide of (1 → 3), (1 → 4)-β-D- that encourages the growth and activity of the colonic bacterial population by behaving like a prebiotic [27]. In addition, β-glucans are used as fat replacers in non-fat and low-fat dairy products for their water binding, thickening, gelling, emulsification, and prebiotic properties [19,28,29,30].

The use of β-glucans in dairy formulations has some limitations because of the phase separation reaction of milk proteins (mainly casein) and β-glucan, known as thermodynamic incompatibility [22,28,29,30,31]. This interaction between casein and β-glucan creates two phases, hindering the physical stability of yogurt and dairy beverages, forming a polysaccharide-rich upper phase and a protein-rich lower phase. The molecular weight of β-glucan preparations and the concentration used are the key factors determining the intensity of phase separation [29] and the use of lower molecular weight β-glucans, as well as within the critical β-glucan concentrations, are recommended solutions. Thermodynamic incompatibility is encountered in the aqueous phase of protein and polysaccharide components due to electrostatic repulsive forces. The high-pressure homogenization (HPH) technique attained an approved effect on emulsion stabilization [32]. Microfluidization, a HPH technique, is promising in terms of yogurt texture and is more effective than conventional homogenization [33,34,35]. HPH causes structural changes in casein micelles that affect the interaction of casein with other constituents and a reduction in the molecular weight of polysaccharides, which is effective for the rheological behavior of polysaccharides in an aqueous system [32]. It provides stable, good-quality viscosity and a creamy structure, especially in low-fat yogurts [33,34,35].

Based on these, in this study, oat β-glucan and reduced fat goat milk were coupled together for the production of physiologically fortified and sensorially improved goat milk yogurt production, and the effect of the microfluidization technique on some quality characteristics of the final product was also observed. The goaty flavor limits goat milk yogurt consumption. Studies have revealed that sensory improvement is achieved in goat milk yogurt by mixing goat milk and cow milk [36], and also through the use of fruit juices, β-cyclodextrins, etc. As a result, in this study, the oat β-glucan was used in different ratios for sensory improvement of goat milk yogurt. In the literature, only one study used oat flour in goat milk yogurt production, observing the textural and organoleptic characteristics [37]. The β-glucan ratios used for yogurt in the literature are as follows: 0.1, 0.2, 0,3, 0.4, and 0.5% [22], 0.25, 0.50, and 1.0% [19], 0.5, 1.0, 1.5, and 2% [38], 0.1, 0.2, and 0.5 [31], 0.2–0.8% [23], 0.5, 1.0, 1.5., 2.0, and 2.5% [39], and 0.1%, 0.2%, 0.3%, 0.4%, and 0.5% [20]. Among these studies, Brennan and Tudorica, in 2008 [39], found that a 0.5% β-glucan ratio was enough for serum retention; Raikos et al., in 2018 [23], observed an adverse flavor at 0.8% β-glucan; Qu et al., in 2021 [20], declared that yogurt samples were sensorially acceptable at up to oat β-glucan ratios of 0.5% and the texture of yogurt samples was not significantly different up to or equal to 0.4%. Additionally, Vasiljevic et al., in 2007 [28], determined that 0.24% and below was inhibitory for phase separation, while other researchers found the threshold for β-glucan concentrations (%, *w*/*w*) phase separation was <0.25% [31], <0.20 [30], and <0.5% [40]. Considering these results and the preliminary experiments carried out; samples with oat β-glucan ratios of more than 0.50% were found to be sensorially unacceptable due to the intense cereal taste and their distinct phase separation. As a result, in the study, the correct oat β-glucan ratios were determined to be 0%, 0.25%, and 0.50%, regarding the sensorial and textural attributes. To the best of our knowledge, in the literature, no other study explored oat β-glucan use in goat milk yogurt and utilized microfluidization for textural improvement in that system. Oat β-glucan-casein interactions are limited in terms of using β-glucans in dairy systems due to thermodynamic incompatibility. From a technological point of view, the results are promising for providing valuable information for developing new-generation dairy systems with β-glucan fortification and providing a new solution with microfluidization for thermodynamic incompatibility reactions faced in β-glucan-casein interactions.

## 2. Materials and Methods

### 2.1. Materials

Raw goat milk was freshly obtained from a dairy farmer in Yeni Pecenek Village of Ankara, Turkey. Skim goat milk powder was supplied by Enka Dairy and Food Products Industry and Commerce Ltd., Co., (Konya, Turkey) and used for the enrichment of the non-fat dry matter content of raw milk. The oat β-glucan product used in the fortification of goat milk yogurt samples with dietary fiber was purchased from the producer (PromOat^®^, Tate & Lyle, Kimstad, Sweden AB). The general characteristics of raw goat milk, skim goat milk powder, and the oat β-glucan product used in yogurt production are presented in Table 1.

### 2.2. Preparation of Milk Blends

Raw goat milk was initially standardized in terms of fat (1.5%) and non-fat solid (16%) for getting physically good quality goat milk yogurt. In the standardization of non-fat solid content, skim goat milk powder (Table 1) was added to approximately 60 °C heated raw goat milk. Milk blends (4000 mL each) were prepared by incorporating different levels of oat β-glucan (0.25% and 0.50% *w*/*v*, on a milk basis), except for control samples. Prepared milk blends were mixed with a laboratory-type Ultra Turax blender (IKA RW 20, Staufen, Germany) at 13,500 rpm for 5 min concerning complete dispersion of the components. Yogurt samples were produced with non-microfluidized and microfluidized milk blends according to the procedure described in the following section. Samples were coded as YC, Y1, and Y2 for non-microfluidized and MFYC, MFY1, and MFY2 for microfluidized yogurt samples (Table 2).

### 2.3. Microfluidization Process and Yogurt Making

Microfluidization was carried out by Microfluidizer (M-110Y, Microfluidics, Westwood, MA, USA) at 103.4 MPa pressure by passing the milk blends of MFYC, MFY1, and MFY2 through the 100 µm process chamber at one stage. Following this, microfluidized milk blends were processed into yogurt. Non-microfluidized samples (YC, Y1, Y2) were directly processed into yogurt. Yogurt production was carried out starting with heat treatment at 90 °C for 10 min and cooling to 43–45 °C for inoculation with mixed lyophilized commercial starter cultures of *Streptococcus thermophilus* and *Lactobacillus delbrueckii* subsp. *bulgaricus* (Y401, Maysa Food Industry and Commerce Ltd. Co., Istanbul, Turkey) at a ratio of 0.4 g/L for each 4 L-batch. After inoculation, all samples were filled in sterile plastic containers of 100 mL volume and incubated at 43 ± 1 °C until the pH value reached 4.6 ± 0.1. At the end of the incubation period, yogurt samples were left to cool for 10 min at room temperature and then stored in refrigerated conditions (4 ± 1 °C). Yogurt samples were analyzed in their physical, chemical, microbiological, textural and sensory properties every seven days of the 21-day storage period.

### 2.4. Proximate Composition of Raw Goat Milk

Raw goat milk was analyzed in basic compositional properties, pH, and titratable acidity as Lactic Acid (LA %). Total dry matter (%) and fat (%) and titratable acidity in Lactic Acid (LA %) by the method [41], ash (%) content was determined by [42]. The pH of raw goat milk was measured by a digital pH meter (Mettler Toledo Seven2Go S2; Schwerzenbach, Switzerland). Kjeldahl method, was used in determining the total protein content of yogurt samples [43]. In analysis, samples were initially digested (Büchi K435, Flawil, Switzerland) and distilled (Büchi 323, Flawil, Switzerland). Total nitrogen (%) was converted to Total Protein (TP %) by multiplying with a factor of 6.38.

### 2.5. Proximate Composition and Physico-Chemical Properties of Yogurt Samples

Yogurt samples were analyzed in total dry matter (%), fat (%), and ash (%) content on the 1st, 7th, 14th, and 21st days of storage according to the methods of [42], Gerber method [41,44,45], respectively. Yogurt samples were diluted with distilled water in a 1:1 ratio, and pH values were measured digitally by a pH meter (Mettler Toledo Seven2Go S2; Schwerzenbach, Switzerland). Titratable acidity values of the yogurt samples were determined and expressed as lactic acid (LA %) [45].

### 2.6. Oat-β-Glucan Content

The β-glucan content of oat-β-glucan fortified yogurt samples was determined by using an enzymatic kit (Megazyme International Ireland Ltd., Co., Wicklow, Ireland) following the Megazyme method and analysis procedure provided by the company [46]. The amount of β-glucan was calculated according to Equation (1). In the enzymatic reaction, the repeated glucose units in the β-glucan structure were broken down into D-glucose by the action of the enzyme β-glucosidase. The absorbance value of the D-glucose released at the end of the lysis reaction was measured at 510 nm wavelength by spectrophotometer (PerkinElmer Lambda 25 UV/Vis Spectrometer, Republic of Singapore). The β-glucan content of yogurt samples was analyzed on the 1st and 21st days of storage. The values obtained for the samples were expressed in g/100 mL (Equation (1)).

(1) $$\beta -glucan\;\left(\frac{g}{100}mL\right)=∆A\times F\times D\times 0.0027$$

$$∆A={Absorbance}_{\;reaction\;}-{Absorbance}_{\;\begin{array}{c} blank \end{array}}$$

$$F=100\left(\mu g\;D-glucose\right)÷({Absorbance}_{\;100\mu gD-glucose\;\;})$$

D=Dilution rate  prior to β−glucosidase enzyme incubation

### 2.7. Color Analysis

Color attributes of yogurt samples were evaluated according to CIELAB color space with the coordinates of L*, a*, and b* denoting lightness/darkness, redness/greenness, and yellowness/blueness, respectively (Konica Minolta CR 410, Sensing Inc., Osaka, Japan). In the coordinates, L* = 100 indicated lightness, and L* = 0 indicated darkness of the sample, (+) a* values indicated redness and (−) a* values indicated greenness and (+) b* values indicated yellowness, and (−) b* values indicated blueness of the sample. After calibrating the instrument with a standard white plate, the instrument measurements were done by putting yogurt samples in a quartz container. The measurements were performed in triplicate.

Color differences among the samples were given by using calculated indices as the total color difference (Δ*E*), Chroma (C*), Hue Angle (h*), Whiteness Index (*WI*) and Yellowness Index (*YI*). In the calculation of these indices the Equations (2)–(6) were used by considering the measured CIE color parameters (L*, a* and b*) of the yogurt samples [47]. The *L*_0_*, *a*_0_* and *b*_0_* values were measured values for control samples (YC and MFYC) and considered as the references for calculation of Δ*E* of non-microfluidized and microfluidized yogurt samples separately (data not given).

(2) $$∆E=\sqrt{∆a^{*2}+∆b^{*2}+∆L^{*2}}$$

(3) $$C^{*}=\sqrt{{a*}^{2}+{b*}^{2}}$$

(4) $$h^{*\;}=\tan^{-1}\left(\frac{{\;b}^{*}}{{a^{*}}^{\;}}\right)$$

(5) $$WI=100-\sqrt{{\left(100-L^{*}\right)}^{2}+a^{*2}+b^{*2}}$$

(6) $$YI=\frac{142.86b^{*}}{L^{*}}$$

### 2.8. Evaluation of Yogurt Gel Properties

#### 2.8.1. Syneresis

Robitaille et al. (2009) [48], determined the syneresis of yogurt samples. Twenty-five grams of yogurt samples were centrifuged for 10 min at 2200 rpm and 4 °C by centrifuge (Sigma 3–18K, Osterode am Harz, Germany). At the end of centrifugation, the supernatant (serum) separated at the top of the tubes was weighed, and syneresis (%) was calculated according to Equation (7).

(7) $$Syneresis\;\left(\%\right)=\left(weight\;of\;supernatant÷weight\;of\;yogurt\right)\times 100$$

#### 2.8.2. Textural Analysis

Back-extrusion method was used in the characterization of yogurt samples’ texture. In the measurements, the texture analyzer (TA. XT Plus, Stable Micro Systems, Surrey, UK) with the equipment of a 5-kg load cell and 35-mm back extrusion disk was used. All samples were analyzed in original containers compatible with the method and equipment. The test conditions were adjusted as: 1.5 mm/s probe test speed and 20 mm for probe penetration distance. Sample temperature is a critical parameter in texture analysis, so samples were removed from the refrigerator (4 ± 1 °C) immediately before the measurement. Quaternary measurements were performed for each sample in the 1st, 7th, 14th, and 21st days of storage. The derived texture attributes from the force-time curve obtained by the back-extrusion method were firmness, consistency, cohesiveness, and index of viscosity.

### 2.9. Culture Viability of Yogurt Bacteria

The growth of yogurt bacteria (*Streptococcus thermophilus* and *Lactobacillus delbrueckii* subsp. *bulgaricus*) in oat β-glucan fortified goat milk yogurt was evaluated during the storage period according to procedures expressed in International Dairy Federation Standard [42,43]. For enumeration of *Lactobacillus delbrueckii* subsp. *bulgaricus* MRS agar medium was used, and incubation was carried out in anaerobic conditions with jars at 37 °C for 72 h for *Streptococcus thermophilus* in M17 agar medium was used. Petri plates were incubated at 37 °C for 48 h aerobically. Yogurt samples were also observed for yeast and mold in PDA (Potato dextrose agar) agar medium at 25 °C for 72–120 h of incubation. Before pouring into the plates, the PDA medium was initially acidified by sterile tartaric acid (10%), 1 mL for each 100 mL PDA agar medium. Plates with 30–300 colonies were recorded, and bacterial counts were evaluated as log CFU × g^−1^. Duplicate examinations were done for all yogurt samples.

### 2.10. Sensory Analysis

Sensory evaluation was conducted with 7-experienced panelists who regularly consumed dairy products in the Department of Dairy Technology, Ankara, Turkey. A scoring guide was prepared with some additional modifications related to criteria encountered in goat milk yogurt and oat-beta glucan (Table 3) [49,50]. In Table 3, panelists were asked to evaluate the yogurt samples over 5 points in terms of color and appearance, structure and texture attributes, degree of liking, and in terms of odor and flavor 10-point rating scale was used. In the evaluation, approximately 50 mL of randomly-coded yogurt sample (in 3-digit numbers) was presented to the panelists with a glass of drinking water in their original containers.

### 2.11. Statistical Analysis

The study was organized by considering the three different levels of oat β-glucan ratio (0%, 0.25% and 0.50%), four different levels of storage time (1, 7, 14 and 21 days) and two different levels of microfluidization conditions (either non-microfluidized or microfluidized) as factors in oat β-glucan fortified reduced fat goat milk yogurt production. This experiment, conducted using the repeated measurement ANOVA technique with oat β-glucan and storage time factors, was replicated in non-microfluidized and microfluidized conditions. The experiment is a repeated-trial conducted using the factorial design with the repeated measurement analysis of variance (ANOVA) method. Statistical analysis of the data was performed using Minitab statistical software (version 16.0, Minitab Inc., State College, PA, USA) and multiple comparisons were made by Duncan test at significance levels of *p* < 0.05 and/or *p* < 0.01.

## 3. Results and Discussion

The proximate composition, physicochemical, textural, and microbiological properties of non-microfluidized (YC, Y1, Y2) and microfluidized (MFYC, MFY1, MFY2) oat β-glucan fortified goat milk yogurt samples in the storage days (1st, 7th, 14th and 21st) were discussed in the whole manuscript by covering the individual effect of oat β-glucan use and its concentration, the individual effect of using microfluidization and the individual effect of storage time on yogurt sample characteristics, and also the effect of interactions of these factors in statistical significance level (*p* < 0.01 and *p* < 0.05) as given in Table 4. For this reason, the results of the parameters for yogurt samples were grouped by factor issues as given in Table 4 and discussed in the relevant sections.

Fat (%) content of yogurt samples was determined as 1.60% and remained un-changed during 21 days of storage. In the manufacture raw goat’s milk was initially standardized to 1.5% fat but fat content of oat β-glucan also contributed to the fat content of the yoghurt samples (Table 1).

### 3.1. Oat β-glucan Content and Effect of Oat-β Glucan Concentration on Some Characteristics of Yogurt Samples

In the inclusion of oat β-glucan, the oat β-glucan with 35% β-glucan were used (Table 1). The amounts incorporated to yogurt samples in ratios of 0.25% and 0.50% were calculated as 0.09 g and 0.18, respectively. However, according to Table 5, yogurt with oat β-glucan 0.25% and oat β-glucan 0.50% contained β-glucan in amounts of 0.05 ± 0.0190 and 0.11 ± 0.0030. These values were lower than inclusion amounts so some amount of β-glucan might have been consumed during fermentation since no change was observed from day 1 to day 21 (data not given). In literature, some researchers declared the utilization of β-glucan by probiotic bacteria and yogurt bacteria resulting in a loss of β-glucan that may weaken the product’s functional properties. So, Gee et al. (2007) [51] and Snart et al. (2005) [52], suggested that the addition of β-glucan should be done after fermentation. Some studies show that selected probiotics and strains of yogurt bacteria can benefit from β-glucan [53]. However, it is not completely clear whether the strains digest β-glucan or whether it has mediating effects for culture growth [28]. Vasiljevic et al. (2007) [28] also concluded that it was unclear whether yogurt starter cultures and probiotics can utilize β-glucan as a prebiotic. However, it was evident that their growth and viability were enhanced. In the studies, no adverse effect of β-glucan use on yogurt bacteria growth rate was declared by researchers [51,54]. Researchers stated that the addition of β-glucan did not affect the growth of yogurt bacteria [28,55,56,57].

According to Table 5, oat β-glucan use and its concentration had a significant effect on dry matter (*p* < 0.01) and similarly on LA %, pH, and index of viscosity values of all samples (*p* < 0.05). Similarly declared as; the dry matter content of oat β-glucan fortified yogurt samples increased relevant to the amount of oat β-glucan used in the samples [19]. The dry matter content of yogurt samples fortified with 0.25% oat β-glucan and 0.50% oat β-glucan (both non-microfluidized and microfluidized) were significantly higher than that of control yogurts (*p* < 0.01) but not found significantly different among yogurt samples fortified with 0.25% oat β-glucan and 0.50% oat β-glucan.

Oat β-glucan use, and its concentration created a significant difference in LA % and pH of all samples (*p* ˂ 0.05). Among the samples, oat β-glucan fortified yogurt with 0.25% concentration was significantly different from control and 0.50% oat β-glucan fortified samples in terms of LA % and again significantly different than 0.50% oat β-glucan fortified samples for pH (Table 5). Sahan et al. (2008) [19] found that using β-glucan composite did not significantly change non-fat yogurt samples’ pH and titratable acidity.

**Figure 1:**
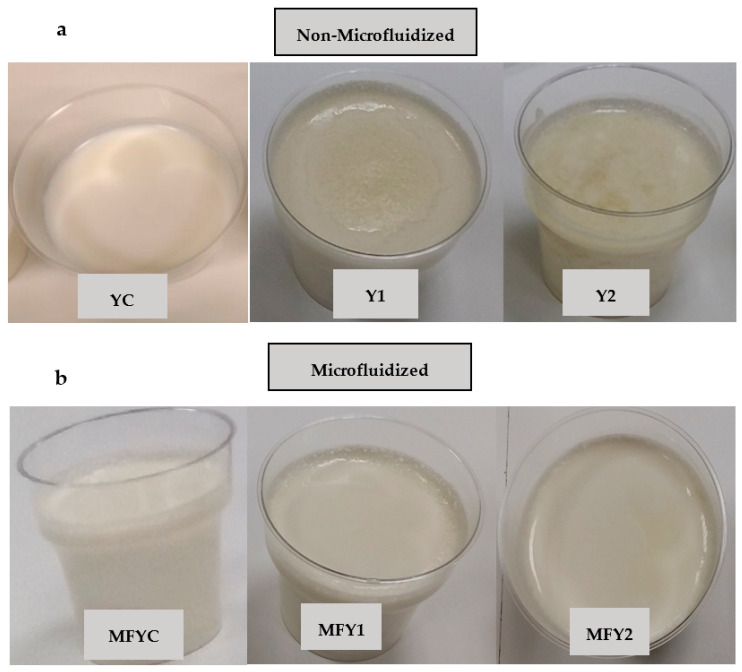
The visual appearance of non-Microfluidized (**a**) and Microfluidized (**b**) oat β-glucan fortified goat milk yogurt samples. YC: non-microfluidized control yogurt (0% oat β-glucan ratio), Y1: non-microfluidized yogurt with 0.25% oat β-glucan ratio, Y2: non-microfluidized yogurt with 0.50% oat β-glucan ratio, MFYC: microfluidized control yogurt (0% oat β-glucan ratio), MFY1: microfluidized yogurt with 0.25% oat β-glucan ratio, MFY2: microfluidized yogurt with 0.50% oat β-glucan ratio.

The index of viscosity measures the sample’s resistance to flow off through the disk during instrumental texture analysis (back extrusion method) [33]. An oat β-glucan ratio-dependent variation was observed for the index of viscosity of yogurt samples. The index of viscosity of yoghurt samples increased when the increase in oat β-glucan ratio was at the level of 0.25%, but a significant decrease was observed at the level of 0.50% (*p* < 0.05) (Table 5). From Table 5, for 0.25% oat β-glucan fortified goat milk yogurt samples an insignificant increase was observed in index of viscosity when compared with control yogurts. In dairy systems with β-glucans above the critical β-glucan level, the casein-β-glucan interaction causes a 2-layer structure resulting in drinkable, low-viscosity yogurts [28,58]. If the emulsion is unstable, the components compromising the system will separate [59]. Stability, viscosity, and water holding capacity are yogurt quality parameters, which are enhanced by homogenization enabling uniform spreading of milk components and ingredients. High-pressure homogenization by microfluidization supplies stable emulsions [33], as it was in our samples (Figure 1).

### 3.2. Impact of Microfluidization and Oat-β Glucan Concentration on Color Attributes, Syneresis, and General Acceptability of Yogurt Samples

Color parameters (L*, a*, and b*) and syneresis (%) of goat-milk yogurt samples were significantly affected by the interaction of oat β-glucan and the microfluidization interaction and given in Table 6. Non-microfluidized samples differed in L* and b* (*p* < 0.01). Among the microfluidized samples, goat-milk yogurt with oat β-glucan 0.50% ratio was significantly different from the others in terms of L* value (less bright) (*p* < 0.01). Lightness decreased, and oppositely yellowness increased (higher b* values) in both non-microfluidized and non-microfluidized samples with increasing amounts of oat β-glucan. As the concentration of oat β-glucan increases b* value increases and the L* value decreases due to the slightly yellowish color of the oat β-glucan product used. Microfluidized control yogurt was significantly different in b* than microfluidized goat-milk yogurt with oat β-glucan 0.50% ratio (*p* < 0.01). In non-microfluidized samples, the intense mass accumulation and serum separation occurred due to an increase in β-glucan concentration, which might decrease L* values and increase yellowness (b*). In oat-based fermented products, control yogurts were whiter than samples with oat β-glucan [60]. Similar results were observed in probiotic yogurts with oat-based and barley-based β-glucan [61]. Including oat β-glucan up to 0.3% had no significant effect on yogurt samples’ lightness (L*). However, a darker color was formed with lower L* values by increasing the concentration of oat β-glucan from 0.3% to 0.5% [22].

The negative a* values indicated that all goat milk yogurt samples (with or without oat β-glucan) were in green color space, and non-microfluidized samples were more greenish than microfluidized ones with higher (−) a* values. As the concentration of oat β-glucan increased, higher a* values were obtained in the negative area. No significant difference was found among microfluidized samples, but in non-microfluidized samples, goat-milk yogurt fortified with 0.50% oat β-glucan ratio was significantly different and more greenish than control and goat-milk yogurt with 0.25% oat β-glucan ratio (*p* < 0.05).

The interaction of oat β-glucan concentration and microfluidization treatment indicated that the lightness (L*) of the samples increased (*p* < 0.01), but a* (greenness) decreased with microfluidization (*p* < 0.05). The increase in lightness among non-microfluidized and microfluidized yogurt groups can be explained by whey protein denaturation and its association with casein micelle induced by pressures of 100–200 MPa applied during homogenization. However, higher pressures (300 MPa) reduce lightness [62]. Similar to our results (Table 6), an increase in lightness was associated with microfluidization application at different pressures for Cheddar cheese [63]. Oppositely, Gervilla et al. (2001) [64], declared that a decrease in L* was caused by the dispersion of casein micelles into smaller pieces resulting from high pressures applied and milk becoming transparent. Microfluidization significantly changed L* (*p* < 0.01) but not for a* and b* for control yogurts. The b* (yellowness) value increased with microfluidization in control yogurt. In goat-milk yogurt fortified with 0.25% oat β-glucan ratio, L* differed statistically (*p* < 0.01) among non-microfluidized and microfluidized samples. Additionally, the difference was not significant for L* but was significant for a* (*p* < 0.05) and b* (*p* < 0.01) among non-microfluidized and microfluidized yogurt samples with 0.5% oat β-glucan (Table 6). Microfluidized samples were less green (a*) than non-microfluidized ones. Lower b* values were measured for only microfluidized goat-milk yogurt fortified with 0.50% oat β-glucan ratio primarily related to homogenization supplied by microfluidization.

According to Table 6, using oat β-glucan in varying amounts and microfluidization treatment created a significant difference in the syneresis of samples (*p* < 0.01). In non-microfluidized samples, syneresis values increased gradually with oat β-glucan concentration and were higher than the control sample. Like ours, syneresis increased at β-glucan levels higher than 0.3% [54]. However, no linear relationship between the amount of β-glucan composite and the syneresis and this time oppositely, syneresis decreased in yogurt samples with β-glucan composite addition [19]. The difference was significant with non-microfluidized control and non-microfluidized oat β-glucan fortified samples (*p* < 0.01) but insignificant among samples with 0.25% and 0.50% oat β-glucan ratio. For microfluidized samples, syneresis values were lower than non-microfluidized couples with similar oat β-glucan content (Table 6). In microfluidized samples, the highest syneresis values were obtained in yogurt samples with 0.5% oat β-glucan ratio, and the lowest was in yogurt samples with 0.25% oat β-glucan ratio, probably due to the more homogeneous distribution of oat β-glucan in the system and the improved water-holding capacity of oat β-glucan (dietary fiber) by microfluidization. However, this behavior did not continue parallel with the increase in oat β-glucan concentration. Although insignificant, higher syneresis was observed for microfluidized yogurt with a 0.50% oat β-glucan ratio (Table 6). According to Lazaridou and Biliaderis (2007) [58], this was due to phase separation due to the interaction between oat β-glucan and casein above a critical concentration level and the thermodynamic incompatibility between these two molecules. Oat and barley β-glucan addition resulted in higher syneresis values obtained in yogurts compared to the control sample and recommended using a β-glucan concentration not higher than 0.4% [28]. At levels up to 0.3% of β-glucan addition, a significant increase in hardness values was observed in yogurt gels with the increase in concentration, and this was found concerning decreased water in the gel structure due to increased syneresis [22].

Oat β-glucan concentration and microfluidization interaction created no significant difference in terms of syneresis among both non-microfluidized and microfluidized control yogurts (*p* ˃ 0.05). However, they were significant (*p* < 0.01) among oat β-glucan fortified non-microfluidized and microfluidized groups (Table 6). In general, in the samples, syneresis (%) decreased by microfluidization (Table 6). The denaturation degree of serum proteins is effective on water holding capacity and the syneresis values of yogurt samples as the denaturation degree of serum proteins increases water holding capacity increases and meaning that syneresis decreases [65,66]. In addition, high-pressure technology causes the casein micelles to break into smaller fragments, dissolution of colloidal calcium phosphate, and denaturation of serum proteins [67,68]. Yogurt texture is improved with increased amounts of reacting denatured serum proteins and the increased number of fat globules and casein micelles [33].

The difference in syneresis of yogurt samples with similar oat β-glucan ratio was correlated with the effect of microfluidization on the functionality of dietary fibers and the increased denaturation ratio of serum proteins. Chen et al., 2013 [69] found that microfluidization treatment increased the solubility of insoluble dietary fibers (peach and oat) by changing the structures of the regions acting on water binding and improving their water-holding capacity. Similarly, Ronkart et al. (2010) [70] stated that microfluidization decreases the particle size of inulin in inulin-water systems independent of the inulin concentration, and the more porous structure is formed by microfluidization and water holding capacity is increased. In our samples (Table 6), the joint effect of microfluidization on denaturation of serum proteins and oat beta-glucan functionality could result in lower syneresis values in microfluidized oat β-glucan fortified yogurt samples (*p* < 0.01).

In sensorial evaluation, the interaction of oat β-glucan concentration and microfluidization effect was found statistically significant in the general acceptability scores of the samples (*p* < 0.01). Non-microfluidized control (YC) and oat β-glucan fortified yogurt with a ratio of 0.25% (Y1) did not differ in terms of general acceptability, but Y2 differed significantly from YC and Y1 (*p* < 0.01). Y1 got the highest score and was followed by YC and Y2 orderly. Increasing oat β-glucan brings many obstacles, especially in dairy systems, due to the interaction of casein and β-glucan. Textural defects are considered significant determinants in yogurt quality, dominating the other favorable properties. As a result, the definite result of using 0.50% oat β-glucan in non-microfluidized samples had an adverse effect on the total quality of goat milk yogurt samples. In microfluidized samples, no significant difference was observed in the general acceptability scores of samples (MFYC, MFY1, and MFY2) (Table 6). MFY1 got the highest score and was followed by MFY2 and MFYC orderly. Fortification concentration of oat β-glucan was found to be statistically significant among the non-microfluidized sample (Y2) and microfluidized sample (MFY2) (*p* < 0.01).

### 3.3. Derived Color Indices of non-Microfluidized and Microfluidized Oat β-Glucan Fortified Goat Milk Yogurt Samples during Storage Period

In Table 7, the results of calculated color indices obtained by the Equations (2)–(6) were given. Δ*E* values were derived by measured values as L*, a* and b* (Equation (2)). The Δ*E*, indicates the color difference among samples and their controls. The degree of the difference was analytically scaled as very distinct difference when Δ*E* > 3, distinct difference when 1.5 < Δ*E* < 3 and a small difference when Δ*E* < 1.5 [47]. From Table 7, in non-microfluidized samples a small difference was observed between YC and Y1 in all storage days with Δ*E* values smaller than 1.5. At ratios of oat β-glucan 0.50%, samples a distinct difference was observed on days of 1st, 7th and 14th (Table 7). In case of microfluidized samples, all samples (MFY1 and MFY2) were had small difference than MFYC. These results can be explained by more homogeneous structure maintained by microfluidization and also more successful dispersion of oat β-glucan and milk constituents in microfluidized samples.

Higher Chroma values meaning that the higher the color in quantity and more intense the color sensed by human eye [47]. The Chroma values increased gradually with oat β-glucan ratio in non-microfluidized and microfluidized yogurt samples but lower values observed for the former. The Chroma values of microfluidized samples increased gradually with oat β-glucan ratio but were nearly constant over time for MFY1 and MFY2. Practically a steady increase was observed for YC, Y1 and Y2 during storage period (Table 7).

Hue angle, defines the qualitative measure of color comparing a certain color with grey color as reference at the same lightness. Angles of 0° and/or 360° defines red hue, 90°, 180°, and 270° defines yellow, green and blue hues in order [47]. The hue angle values of all oat β-glucan fortified samples were in yellow/green color region but mostly yellow with angles very close to 90°. The hue angle of non-microfluidized samples were higher but with a very small margin than microfluidized samples. The hue angles of microfluidized samples were very close to each other presented nearly a constant trend in storage period (Table 7).

Whiteness index, indicates the degree of whiteness of a product in general [47]. The highest whiteness index values were observed for microfluidized samples. Among the samples YC and MFYC got highest whiteness index values. Inclusion of oat β-glucan resulted in a gradual decrease but with nearly 2 units among Y1 and Y2 (more than MFY1 and MFY2) possibly related to effect of homogeneous distribution of oat β-glucan by microfluidization (Table 7).

Yellowness index, changes by processing, exposure to light and chemicals and explains the degree of yellowness [47]. A gradual increase was observed in yellowness index values of non-microfluidized samples with nearly 2 units. Similar trend was observed in microfluidized samples but this time with small increments. Yellowness increased was initially thought to be associated with oat β-glucan inclusion and its ratio first, but microfluidized samples also presented a homogeneity in values in general that were lower than Y1 and Y2 (Table 7).

### 3.4. Effect of Microfluidization and Storage Time on Some Physico-Chemical, Proximate Composition, and Textural Characteristics of Yogurt Samples

The interaction of microfluidization and storage time was found significant in terms of dry matter, lactic acid, ash, and a* (*p* < 0.01) and for pH, L*, syneresis, and cohesiveness (*p* < 0.05) (Table 8). Non-microfluidized samples did not differ significantly in dry matter and ash in all storage time (*p* > 0.05), but non-microfluidized samples were different from microfluidized samples in terms of dry matter and ash (means ± SE) on all storage days (*p* < 0.01). In non-microfluidized and microfluidized samples, pH was significantly changed during the storage period, with no change on day 7 and day 14 (Table 8). However, among the groups (non-microfluidized and microfluidized), the pH changes on the 7th, 14th, and 21st days was significant (*p* < 0.05). In the samples on the 1st day, means ± SE of titratable acidity and pH values of non-microfluidized samples were lower than (less acidic) microfluidized ones. A gradual increase was observed in LA % of all yogurt samples, and from day 1 to day 21, these changes were 0.18 and 0.06 units for non-microfluidized and microfluidized samples, respectively (Table 8). During storage, yogurt samples’ pH values gradually decreased [71,72]. According to Table 8, the pH drop in the samples was more pronounced between 1st and 7th days of storage and similar to results obtained it was associated with the high rate of lactose consumption and higher production of lactic acid and galactose in these days [73]. Prebiotics in apple pomace powder nourished lactic acid bacteria and acted in additional lactic acid production [72]; similar can be concluded for oat β-glucan fortified yogurt samples. High-pressure homogenization has an inhibitory effect on the growth of yogurt bacteria and *Lactobacillus delbrueckii* subsp. *bulgaricus* and stimulate the *Streptococcus thermophilus* [34]. The exposure of microfluidized milk blends to an additional treatment in which a slight temperature increase occurred during microfluidization could be responsible for this difference. The initial pH of both groups of yogurt samples ranged from 4.3–4.41, similar to Herrero and Requena (2006) [74], which ranged from 4.31–4.48. The activity of yogurt starter cultures *L. delbrueckii* subsp. *bulgaricus* and *Streptococcus thermophilus* continues during refrigerated conditions, and acidity increases [75]. The post-acidification reaction of yogurt starter cultures is responsible for the pH drop during the storage period [76,77,78] and related to β-galactosidase activity that is active at 0–5 °C is very closely linked to the drop of pH values lower than 4.2. At this stage survival degree of lactic acid bacteria is adversely affected, and syneresis is observed [75]. From Table 8, the pH values of all samples were higher than this limit indicating over-acidification.

The L* values increased in non-microfluidized samples, and the first day’s L* values differed significantly from the other storage days (*p* < 0.05) (Table 8). L* of microfluidized samples remained almost constant (*p* ˃ 0.05), possibly due to supplied homogeneity and stability in the samples. Both non-microfluidized and microfluidized samples got negative a* values throughout the storage period, indicating they exist in green color space. The a* values increased greenness and became more intense from day 1 to day 21 in non-microfluidized samples, with a significant increase on the 21st day. Non-microfluidized samples were more greenish than microfluidized ones and significant only on the 21st day (*p* < 0.01). In microfluidized samples, similar to L*, a* remained nearly constant during storage (*p* ˃ 0.05) (Table 8).

According to microfluidization and storage time interaction, the syneresis of non-microfluidized samples was higher than microfluidized samples on all storage days (*p* < 0.05). Microfluidization showed superiority in preventing syneresis in oat β-glucan fortified goat milk yogurt systems (Table 8). Syneresis is a textural defect considered a problem in set-type yogurt. It is especially possible in non-fat, low-fat, and low-dry matter yogurt systems [79]. Casein content is known to be more effective on syneresis than serum proteins. In the samples, casein-β-glucan interaction supported syneresis and presented a significant two-layered structure in increasing β-glucan concentrations. However, all yogurt samples got similar fat content but different dry matter content due to oat β–glucan fortification (Table 5). This time microfluidization created a difference among yogurt samples. Syneresis decreased by microfluidization and increased by storage time, related to increased acidity (Table 8). Microfluidization served uniform distribution of oat β-glucan and enhanced water holding capacity of oat β-glucan that resulted in lower syneresis (Table 8). The researchers observed a decrease in syneresis in yogurt during storage [80,81]. Syneresis decreased by storage, especially in cow’s milk yogurt samples with β-glucan composite [19]. However, the reverse was valid for the samples and can be explained by decreased pH values (Table 8).

In non-microfluidized samples, cohesiveness increased from day 1 to day 21 and was statistically significant on the first day than the other days (*p* < 0.05). In microfluidized samples, cohesiveness remained nearly the same during storage (*p* ˃ 0.05). A significant difference was observed between the non-microfluidized and microfluidized samples on the 7th and 21st day (Table 8). In the back-extrusion method on a force-time graph, cohesiveness is the maximum negative force, and it determines the resistance of the samples to the moving disk going away from the sample [33]. As the strength of the internal bonds increases, internal stickiness increases [82]. Microfluidization maintains a more homogeneous and creamy texture, but this is not related to the fat content; it defines the smoothness of the product [83]. In low-fat yogurts, microfluidization improves the texture in that creamy, softness, enhanced density, and viscosity in mouthfeel are supplied [35]. Lower cohesiveness values in microfluidized yogurt samples can depend on the increased smoothness and creaminess (Table 8). Opposite to ours, firmness, elasticity, adhesiveness, and gumminess of milk gels can be enhanced, but cohesiveness can be diminished by β-glucan with specific molecular characteristics [84]. Cohesiveness values of microfluidized yogurts with 0.50% oat β-glucan were higher than non-microfluidized candidates due to the homogeneous structure formed by microfluidization since in non-microfluidized samples with 0.50% oat β-glucan concentration a distinct phase separation was observed due to thermodynamic incompatibility of casein and oat β-glucan. Although further research is required, high-pressure homogenization mainly causes differences in gel microstructure rather than texture in yogurt samples [85].

### 3.5. Effect of Storage Time on Color, Microbiology, and Texture Attributes of Yogurt Samples

Storage time effect was found significant on the parameters for all non-microfluidized and microfluidized goat milk yogurt samples and given in Table 9. The b* value of the samples differed significantly on the first day of storage (*p* < 0.05) and increased gradually up to day 14 with no significant change in the following days of storage (*p* > 0.05). Similarly, b* values of apple fiber-added strained yogurts increased significantly during the 21-day storage period [13].

Firmness values changed significantly on the 1st and 14th, and 21st days of storage (*p* < 0.01) and increased from day 1 to day 21. In dietary fiber-added strained yogurt samples, firmness increased with storage time. It was explained by the increased water-holding capacity of milk proteins and dietary fibers during storage [13]. Similar to firmness values, the consistency of yogurt samples increased slightly during the storage period, and it was significantly different on the 1st day of storage than in the remaining periods (*p* < 0.05) (Table 9). Consistency is a measure of the thickness of samples [33]. Protein rearrangement occurred during storage, increasing firmness and consistency [71].

The viability of the yogurt starter culture presented a storage time-dependent trend with statistical significance (*p* < 0.01). The health benefits of yogurt can be observed when the live bacteria level is 10^8^–10^9^ CFU/g [65]. According to Table 9, *Streptococcus thermophilus* increased in number of colonies until day 7 significantly (*p* < 0.01), but no significant difference was observed on the 14th day and 21st day compared to the 1st day. Table 9 shows a slight decrease in colonies from day 7 to 21. *Lb. delbrueckii* subsp. *bulgaricus* showed a gradual decrease in the number of colonies during storage (*p* < 0.01) (Table 9). The inhibitory effect of high-pressure homogenization on the growth of yogurt bacteria was determined by Lanciotti et al. (2004) [34]. Consistent with the results of Gee et al. (2007) [51] and Valerie (2009) [54], Lanciotti et al. (2004) [34] declared that high-pressure homogenization stimulates the growth of *S. thermophilus* than *Lb. delbrueckii* subsp. *bulgaricus* independent of the applied pressure values. Similarly, during the cold storage of yogurt, the number of *L. delbrueckii* subsp. *bulgaricus* colonies decreased over time; a more pronounced decrease was observed in high-pressure processed milk used in yogurt production (at 200 MPa pressure (30 °C or 40 °C), and this situation was found in relation with the accumulation of pyruvate having a toxic effect on the starter bacteria [86].

### 3.6. Sensory Evaluation

**Figure 2:**
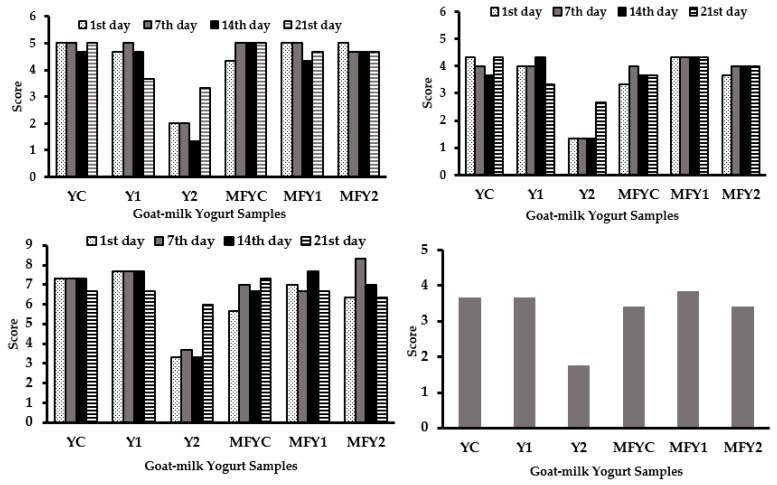
Scores of sensory parameters (Color/appearance (top left), body/texture (top right), taste/flavor (bottom left), general acceptability (bottom right) for non-microfluidized and microfluidized oat β-glucan fortified goat milk yogurt samples (values are the means of the treatments), (n = 3). YC: non-microfluidized control yogurt (0% oat β-glucan ratio), Y1: non-microfluidized yogurt with 0.25% oat β-glucan ratio, Y2: non-microfluidized yogurt with 0.50% oat β-glucan ratio, MFYC: microfluidized control yogurt (0% oat β-glucan ratio), MFY1: microfluidized yogurt with 0.25% oat β-glucan ratio, MFY2: microfluidized yogurt with 0.50% oat β-glucan ratio.

The sensory evaluation results of non-microfluidized (YC, Y1, and Y2) and microfluidized (MFYC, MFY1, and MFY2) oat β-glucan fortified goat milk yogurt samples were shown in Figure 2 for the storage period of 21 days. In terms of color/appearance (top left), among the non-microfluidized group YC (control) and Y1 got very close scores (almost 5 points) except Y2 during the 1st, 7th, and 14th days of storage. On day 21, YC was evaluated with the highest and Y2 with the lowest score. In sensory evaluation, no change was pronounced in the color/appearance of the control sample during the storage period. According to panelists’ verbal definitions, the control sample (YC) was whiter in color than the others, and yellowness increased in the order of Y1 and Y2 depending on the oat β-glucan concentration.

No apparent texture defect was detected in Y1, and very little serum accumulation on the surface of the yogurt was observed. Y2 was the least favored sample on all storage days regarding all sensory parameters (Figure 2). From the 1st day of storage, a definite phase separation (a two-layered structure) was observed in Y2. The serum separation and viscosity of yogurt are essential in its sensorial acceptability [87]. Syneresis increased in yogurts above the 0.3% β-glucan level (critical β-glucan level), and the color darkened compared to the control sample [22]. Y1 had a lower amount of oat β-glucan than the reported critical concentration, so the phase separation was not so apparent. However, in Y2, a very distinct two-layered structure was observed (Figure 1).

In general, microfluidized samples were superior to non-microfluidized samples with more homogeneous scores (Figure 2, top left). Microfluidization treatment improved the samples both texturally and also in color/appearance properties (Figure 2). The microfluidized samples MFYC, MFY1, and MFY2 were improved in color and appearance by homogenization effect acting on increased lightness (L*). In particular, the two-layered phase separation problem occurred due to increasing oat β-glucan concentration was avoided entirely by microfluidization (Figure 1). In terms of color/appearance, MFY2 was evaluated with higher scores than Y2 in all days of storage (Figure 2).

YC and Y1 got higher scores in texture among non-microfluidized samples, and Y2 was the least liked sample in terms of texture (Figure 2). YC got a typical yogurt gel structure but was slightly weak due to goat milk characteristics. Goat’s milk is similar to cow’s milk in total dry matter, protein, fat, and lactose levels [3,81]. However, casein micelles differ significantly in structure, composition, and size, leading to differences in physicochemical properties [81]. Goat milk contains higher levels of non-protein nitrogen and mineral substances and a higher proportion of small-diameter fat globules [3,81]. Qualitative and quantitative differences exist in proteins, especially αs1-casein [1]. Fat content and fat globule size affect the gel firmness. Goat milk yogurts are characterized by soft consistency, low viscosity, low firmness, weak gel structure, and syneresis susceptibility when compared with cow’s milk [5,6,7,8,9,88]. In non-microfluidized samples, the panelists reported phase separation defects becoming intense with increasing concentrations of oat β-glucan (Figure 1). By the way, this resulted in lower scores in Y2 (Figure 2, top right). Y1 exhibited an excellent mouth feel, a non-granular but lumpy texture with a pudding-like structure. According to sensorial texture, Y1 was not thin as YC; besides that, firmer than YC. Panelists reported a significant phase separation in Y2 due to thermodynamic incompatibility between milk proteins and β-glucan. Excessive serum separation, weak gel structure, granular texture, drinkable consistency, and an untypical gel structure far from yogurt texture are some verbal definitions of defects observed for Y2 during storage (Table 3). Similar to that, researchers determined significant syneresis in yogurt samples with 0.50% oat β-glucan product, so they gathered lower texture scores [28].

In general, microfluidization improved the textural properties of all samples, also measured instrumentally. However, as expected MFYC sample was thinner in structure than MFY1 and MFY2 related to goat milk’s weak textural characteristics. A creamy structure was observed in MFYC, MFY1 and MFY2. This creaminess created lower firmness values in MFYC than in YC (data not given). The creamy texture was regarded as a positive consequence of microfluidization on texture. In a study conducted by Brennan and Tudorica (2008) [39], it was revealed that the creamy texture of the samples containing 0.5% and 1.5% β-glucan increased with microfluidization. Similarly, in high-pressure applied samples, the creamy structure became dominant [35,83,89]. Microfluidized oat β-glucan fortified goat milk yogurt samples got higher scores than the control of this group, and MFY1 was evaluated with the highest and identical scores during storage days (Figure 2). Increasing concentrations of oat β-glucan created a structure different than yogurt gel and defined as pudding-like structure and besides that phase separation defect was entirely prevented by microfluidization. However, in microfluidized samples, firmness decreased in the order of MFY1, MFY2, and MFYC (data not given). MFY2 samples were firmer than MFYC due to the joint effect of water-holding capacity oat β-glucan as a dietary fiber and microfluidization.

Regarding taste/flavor, yogurt samples were evaluated over 10 points. Among the non-microfluidized samples, Y1 was the most liked sample with constant scores on the 1st, 7th, and 14th days, and Y2 was the least liked on almost all days. In non-microfluidized oat β-glucan fortified samples, it was advised to consume after stirring due to phase separation defect, especially in Y2, since the two-layered structure affected the taste perception. In oat β-glucan dairy systems, a two-layered structure is formed due to thermodynamic incompatibility, with the upper phase being polysaccharide-rich and the lower phase being protein-rich [28]. A goaty and salty taste was generally pronounced in YC due to goat milk origin. Goat’s milk is characterized by a unique goaty and salty taste/flavor [12] and in the samples of Y1 and Y2, cereal flavor suppressed the salty and goaty taste and the typical yogurt flavor. Typical yogurt flavor and acidity were detected mainly in YC. Grain flavor dominated with increasing concentrations of oat β-glucan.

Like other sensory attributes, the taste/flavor of oat β-glucan fortified goat milk yogurt samples were improved by microfluidization. A noticeable improvement in taste and flavor was maintained in the 0.50% oat β-glucan fortified sample (MFY2). Taste/flavor scores indicated fluctuation during shelf life; the highest scores were obtained for MFYC on the 1st day, for MFY2 on the 14th day, and MFY2 on the 7th day (Figure 2). Oat fiber use in plain yogurt improved texture but decreased flavor attributes in plain yogurt [90] According to a study, in the samples with 0.5% and 1.5% β-glucan, a creamy texture and a soft feeling were maintained on the palate with no election among the samples [39]. The use of β-glucan hydrocolloidal composite in ratios of 0.25% or 0.50% in non-fat yogurts was satisfying, and these were evaluated with scores close to the control that was the most preferred sample [19]. As previously discussed in the related section, in terms of general acceptability, Y2 was the most significantly different sample in the non-microfluidized group and also in its microfluidized couple (*p* < 0.01) (Table 6). To sum up, since the microfluidization process improved the overall quality of the samples and eliminated the physical defects that occurred in the yogurt sample due to oat β-glucan existence, it is possible to say that the general acceptability of microfluidized oat β-glucan fortified samples was higher than their non-microfluidized candidates.

## 4. Conclusions

The study was designed to observe the compatibility of oat β-glucan and goat milk in yogurt system and together with observing the effect of microfluidization treatment in this system. Oat β-glucan fortification can suppress goaty and salty taste of goat milk yogurt so could increase the acceptability depending on the inclusion ratio. Sensorial and textural improvement can be maintained by microfluidization in goat milk yogurt samples independent on oat β-glucan ratio but oat β-glucan ratio could be determinative on sensorial and textural properties of non-microfluidized yogurt samples. It could be concluded that microfluidization can be successfully used in oat β-glucan fortified dairy systems for preventing phase separation occurred due to thermodynamic incompatibility reaction between casein and oat β-glucan.

## Figures and Tables

**Table 1:** General characteristics of raw goat milk, skim goat milk powder, and oat β-glucan.

Component (%)	Raw Goat Milk ^a^	Skim Goat Milk Powder ^b^	Oat β-glucan ^c^
Total solid	14.26 ± 0.83	96.61	96.97
Fat	5.13 ± 0.56	0.50	0.75
Total protein	4.51 ± 0.30	- ^d^	4.25
Ash	0.78 ± 0.06	8.79	2.09
pH	6.67 ± 0.03	6.66	- ^d^
Titratable acidity, % LA	8.37 ± 0.62	0.11	- ^d^
β–glucan	- ^e^	- ^e^	35.00

^a^ Values are means ± standard error of parameters (n = 3). ^b^,^c^ Data supplied by the manufacturer. ^d^ Not determined. ^e^ Not included.

**Table 2:** Details on milk blends for yogurt samples.

	Details for Milk Blends			
Sample Code ^1^	Oat β Glucan Ratio (%, *w*/*v*, on Milk Base)			Microfluidization (MF)
	0	0.25	0.50	
YC	✓	-	-	-
Y1	-	✓	-	-
Y2	-	-	✓	-
MFYC	✓	-	-	✓
MFY1	-	✓	-	✓
MFY2	-	-	✓	✓

^1^ Sample codes denote: YC: non-microfluidized control yogurt (0% oat β-glucan ratio), Y1: non-microfluidized yogurt with 0.25% oat β-glucan ratio, Y2: non-microfluidized yogurt with 0.50% oat β-glucan ratio, MFYC: microfluidized control yogurt (0% oat β-glucan ratio), MFY1: microfluidized yogurt with 0.25% oat β-glucan ratio, MFY2: microfluidized yogurt with 0.50% oat β-glucan ratio.

**Table 3:** Scoring guide for sensory evaluation of yogurt samples.

Quality Criteria	Possible Defects in Goat Milk Yogurt
Color and appearance The score ranges from 1–5 points 5: defines no defect	Non-uniform color
	Free whey
	Unnatural color
	Shrinkage
	Surface growth of bacteria
Structure and texture attributes The score ranges from 1–5 points 5: defines no defect	Too thin or too firm
	Creamy Drinkable Syneresis
	Lumpy
	Granular
	Ropy
Flavor and Odor The score ranges from 1–10 points 10: defines no defect	Lack of yogurt taste and aroma
	Cooked flavor
	Creamy
	Cereal flavor
	Goaty flavor and odor
	Fermented
	Sour
	Salty
	Sweet
	Yeasty/Fruity
	Foreign

**Table 4:** Demonstration of *p* values; individual effect of oat β-glucan use, storage time, microfluidization and their mutual interactions.

Parameter	ANOVA						
	*P*-Oat β-Glucan	*P*-Storage Time	*P*-MF	*P*-MF × *P*-β-Glucan	*P*-MF × *P*-Storage Time	*P*-Oat β-Glucan × *P*-Storage Time	*P*-Oat β-Glucan × *P*-Storage Time × *P*-MF
Dry matter (%)	**	NS	NS	NS	**	NS	NS
Lactic Acid (%)	*	NS	NS	NS	**	NS	NS
pH	*	NS	NS	NS	*	NS	NS
Ash (%)	NS	NS	NS	NS	**	NS	NS
Oat β-glucan	**	NS	NS	NS	NS	NS	NS
Syneresis (%)	NS	NS	NS	**	*	NS	NS
Firmness (N)	NS	**	NS	NS	NS	NS	NS
Consistency (N×s)	NS	*	NS	NS	NS	NS	NS
Cohesiveness (N)	NS	NS	NS	NS	*	NS	NS
Index of viscosity (N×s)	*	NS	NS	NS	NS	NS	NS
L*	NS	NS	NS	**	*	NS	NS
a*	NS	NS	NS	*	**	NS	NS
b*	NS	*	NS	**	NS	NS	NS
*S. thermophilus* (log CFU/g)	NS	**	NS	NS	NS	NS	NS
*Lb. delbrueckii* subsp. *bulgaricus* (log CFU/g)	NS	**	NS	NS	NS	NS	NS
General acceptability	NS	NS	NS	**	NS	NS	NS

* *p* < 0.05, ** *p* < 0.01; NS, not significant *p* > 0.05, *P*-oat β-glucan, individual effect of oat β-glucan, *P*-storage time, individual effect of storage time, *P*-MF, individual of effect of microfluidization, *P*-MF × *P*-β-glucan, interaction between microfluidization and oat β-glucan, *P*-MF × *P*-storage time, interaction between microfluidization and storage time, *P*-oat β-glucan × *P*-storage time, interaction between oat β-glucan and storage time, *P*-oat β-glucan × *P*-storage time × *P*-MF, interaction between oat β-glucan, storage time and microfluidization.

**Table 5:** The individual effect of oat β-glucan concentration in some characteristics of both non-microfluidized and microfluidized goat milk yogurt samples.

Parameter	$\bar{X}$ ± *S_x_* ^1^		
	Control	Oat β-Glucan 0.25%	Oat β-Glucan 0.50%
Dry matter (%)	15.91 ± 0.1100 ^B^**	16.21 ± 0.1290 ^A^**	16.44 ± 0.1300 ^A^**
LA (%)	1.51 ± 0.0155 ^B^*	1.56 ± 0.0127 ^A^*	1.49 ± 0.0170 ^B^*
pH	4.29 ± 0.0196 ^AB^*	4.25 ± 0.0181 ^B^*	4.33 ± 0.0180 ^A^*
Oat β-glucan content (g/100 mL) ^2^	-	0.05 ± 0.0190 ^B^**	0.11 ± 0.0030 ^A^**
Index of viscosity (N × s)	1.13 ± 0.0668 ^A^*	1.17 ± 0.0460 ^A^*	0.70 ± 0.0442 ^B^*

^1^ Data are the means ± SE (n = 24) of parameters for both goat milk yogurt samples (non-microfluidized and microfluidized) with the same oat β-glucan content during the 21-day storage period; ^2^ The values are the means ± SE (n = 12) of oat β-glucan content of both non-microfluidized and microfluidized goat milk yogurt samples on days of 1 and 21. Control: Means of both non-microfluidized and microfluidized control yogurts (YC and MFYC). Oat β-glucan 0.25%: Means of both non-microfluidized and microfluidized yogurts with 0.25% oat β-glucan ratio (Y1 and MFY1), Oat β-glucan 0.50%: Means of both non-microfluidized and microfluidized yogurt with 0.50% oat β-glucan ratio (Y2 and MFY2), different capital letters in the same row indicate significant difference (** *p* < 0.01, * *p* < 0.05).

**Table 6:** Color attributes, syneresis (%), and general acceptability of oat β-glucan fortified goat milk yogurt samples depend on microfluidization and oat β-glucan concentration.

Parameter	Yogurt Sample	$\bar{X}$ ± *S_x_* ^1^	
		Non-Microfluidized	Microfluidized
L*	Control	69.63 ± 0.3980 ^Ab^**	70.30 ± 0.0832 ^Aa^**
	Oat β-glucan 0.25%	68.76 ± 0.3830 ^Bb^**	70.08 ± 0.0989 ^Aa^**
	Oat β-glucan 0.50%	67.26 ± 0.3590 ^Ca^**	69.46 ± 0.1540 ^Ba^**
a*	Control	−2.12 ± 0.1050 ^Aa^*	−2.08 ± 0.0814 ^Aa^*
	Oat β-glucan 0.25%	−2.23 ± 0.1720 ^Aa^*	−2.00 ± 0.0551 ^Aa^*
	Oat β-glucan 0.50%	−2.70 ± 0.1160 ^Bb^*	−2.15 ± 0.0786 ^Aa^
b*	Control	7.90 ± 0.3750 ^Ca^**	8.31 ± 0.0889 ^Ba^**
	Oat β-glucan 0.25%	8.52 ± 0.4060 ^Ba^**	8.53 ± 0.0535 ^ABa^**
	Oat β-glucan 0.50%	9.26 ± 0.3300 ^Aa^**	8.80 ± 0.0484 ^Ab^**
Syneresis (%)	Control	12.53 ± 0.434 ^Ba^**	12.81 ± 0.969 ^Aa^**
	Oat β-glucan 0.25%	21.88 ± 0.853 ^Aa^**	10.34 ± 0.785 ^Ab^**
	Oat β-glucan 0.50%	24.59 ± 2.280 ^Aa^**	15.08 ± 1.400 ^Ab^**
General acceptability	Control	3.62 ± 0.125 ^Aa^**	3.27 ± 0.163 ^Aa^**
	Oat β-glucan 0.25%	3.71 ± 0.132 ^Aa^**	3.83 ± 0.122 ^Aa^**
	Oat β-glucan 0.50%	1.80 ± 0.210 ^Bb^**	3.49 ± 0.168 ^Aa^**

^1^ Data are the means ± SE (n = 12) of parameters for oat β-glucan fortified goat milk yogurt samples during a 21-day storage period; Control: Means of goat-milk yogurt with 0% oat β-glucan ratio either non-microfluidized or microfluidized, Oat β-glucan 0.25%: Goat-milk yogurt fortified with 0.25% oat β-glucan ratio either non-microfluidized or microfluidized, Oat β-glucan 0.50%: Goat-milk yogurt fortified with 0.50% oat β-glucan ratio either non-microfluidized or microfluidized, different capital letters in the same column indicate significant difference (* *p* < 0.05, ** *p* < 0.01), different lower case letters in the same row indicate significant difference (* *p* < 0.05, ** *p* < 0.01).

**Table 7:** Change in color indices of non-Microfluidized and Microfluidized oat β-glucan fortified goat milk yogurt samples during storage period.

Color Indices	Sample	Non-Microfluidized				Sample	Microfluidized			
		Storage Time (Day)					Storage Time (Day)			
		1	7	14	21		1	7	14	21
ΔE	YC	-	-	-	-	MFYC	-	-	-	-
	Y1	1.13 ± 0.17	1.02 ± 0.13	1.13 ± 0.08	1.34 ± 0.09	MFY1	0.38 ± 0.06	0.33 ± 0.06	0.42 ± 0.08	0.46 ± 0.07
	Y2	2.12 ± 0.20	2.15 ± 0.04	1.75 ± 0.05	1.21 ± 0.26	MFY2	0.89 ± 0.24	0.94 ± 0.17	0.63 ± 0.04	0.44 ± 0.13
Chroma	YC	7.06 ± 1.42	8.45 ± 0.08	8.55 ± 0.16	8.69 ± 0.21	MFYC	8.44 ± 0.08	8.63 ± 0.11	8.67 ± 0.19	8.54 ± 0.17
	Y1	7.66 ± 1.51	9.19 ± 0.02	9.09 ± 0.12	9.39 ± 0.13	MFY1	8.66 ± 0.03	8.76 ± 0.08	8.84 ± 0.11	8.84 ± 0.10
	Y2	8.73 ± 1.25	9.99 ± 0.09	10.07 ± 0.16	9.85 ± 0.11	MFY2	8.97 ± 0.13	9.04 ± 0.05	9.17 ± 0.11	9.05 ± 0.03
Hue Angle	YC	107.55 ± 4.40	103.79 ± 0.65	103.46 ± 0.20	106.98 ± 1.72	MFYC	104.24 ± 1.52	104.00 ± 1.29	103.95 ± 1.67	104.20 ± 1.78
	Y1	107.52 ± 5.70	102.55 ± 1.45	103.33 ± 1.79	107.42 ± 2.94	MFY1	103.32 ± 0.96	103.16 ± 0.94	103.09 ± 0.91	103.02 ± 1.07
	Y2	107.43 ± 3.16	105.03 ± 0.92	104.65 ± 0.55	108.64 ± 2.09	MFY2	103.94 ± 0.96	103.63 ± 0.91	103.55 ± 1.28	103.69 ± 1.56
Whiteness Index	YC	67.64 ± 1.18	68.83 ± 0.24	68.46 ± 0.45	69.12 ± 0.09	MFYC	69.16 ± 0.06	69.16 ± 0.28	69.09 ± 0.27	68.96 ± 0.16
	Y1	66.59 ± 1.10	68.02 ± 0.16	67.53 ± 0.28	67.88 ± 0.11	MFY1	68.97 ± 0.15	68.87 ± 0.25	68.74 ± 0.26	68.70 ± 0.26
	Y2	64.77 ± 0.79	65.92 ± 0.19	65.92 ± 0.24	66.74 ± 0.34	MFY2	68.15 ± 0.39	67.99 ± 0.45	68.15 ± 0.22	68.29 ± 0.16
Yellowness Index	YC	13.99 ± 2.85	16.74 ± 0.10	17.05 ± 0.24	16.84 ± 0.29	MFYC	16.61 ± 0.27	16.98 ± 0.37	17.09 ± 0.55	16.84 ± 0.50
	Y1	15.37 ± 3.19	18.45 ± 0.16	18.34 ± 0.40	18.42 ± 0.05	MFY1	17.14 ± 0.09	17.37 ± 0.26	17.56 ± 0.35	17.57 ± 0.34
	Y2	18.01 ± 2.59	20.43 ± 0.17	20.64 ± 0.35	19.51 ± 0.43	MFY2	17.90 ± 0.08	18.11 ± 0.12	18.33 ± 0.37	18.04 ± 0.21

YC: non-microfluidized control yogurt (0% oat β-glucan ratio), Y1: non-microfluidized yogurt with 0.25% oat β-glucan ratio, Y2: non-microfluidized yogurt with 0.50% oat β-glucan ratio, MFYC: microfluidized control yogurt (0% oat β-glucan ratio), MFY1: microfluidized yogurt with 0.25% oat β-glucan ratio, MFY2: microfluidized yogurt with 0.50% oat β-glucan ratio.

**Table 8:** Some physicochemical, proximate composition, and textural characteristics of goat milk yogurt samples depend on microfluidization and storage time.

Parameter	Storage Period (day)	Sample	
		$\bar{X}$ ± *S_x_* ^1^	
		Non-Microfluidized	Microfluidized
Dry matter (%)	1	16.73 ± 0.0923 ^Aa^**	15.58 ± 0.1620 ^Bb^**
	7	16.62 ± 0.1160 ^Aa^**	15.92 ± 0.2050 ^Ab^**
	14	16.63 ± 0.1010 ^Aa^**	15.60 ± 0.1300 ^Bb^**
	21	16.66 ± 0.1300 ^Aa^**	15.75 ± 0.1630 ^ABb^**
Lactic Acid (%)	1	1.41 ± 0.0266 ^Ca^**	1.46 ± 0.0125 ^Ba^**
	7	1.53 ± 0.0250 ^Ba^**	1.53 ± 0.0090 ^Aa^**
	14	1.56 ± 0.0195 ^ABa^**	1.54 ± 0.0096 ^Aa^**
	21	1.59 ± 0.0175 ^Aa^**	1.52 ± 0.0277 ^Ab^**
pH	1	4.41 ± 0.0186 ^Aa^*	4.37 ± 0.0247 ^Aa^*
	7	4.33 ± 0.0287 ^Ba^*	4.21 ± 0.0147 ^BCb^*
	14	4.29 ± 0.0166 ^BCa^*	4.24 ± 0.0250 ^Bb^*
	21	4.26 ± 0.0173 ^Ca^*	4.19 ± 0.0187 ^Cb^*
Ash (%)	1	1.40 ± 0.0104 ^Aa^**	1.09 ± 0.0482 ^Bb^**
	7	1.39 ± 0.0105 ^Aa^**	1.25 ± 0.0080 ^Ab^**
	14	1.40 ± 0.0047 ^Aa^**	1.28 ± 0.0044 ^Ab^**
	21	1.40 ± 0.0074 ^Aa^**	1.28 ± 0.0062 ^Ab^**
*L*	1	67.34 ± 0.7740 ^Bb^*	69.99 ± 0.1940 ^Aa^*
	7	68.93 ± 0.4020 ^Ab^*	69.93 ± 0.2400 ^Aa^*
	14	68.64 ± 0.3660 ^Ab^*	69.95 ± 0.1650 ^Aa^*
	21	69.29 ± 0.3320 ^Aa^*	69.91 ± 0.1210 ^Aa^*
*a*	1	−2.14 ± 0.1170 ^Aa^**	−2.08 ± 0.0901 ^Aa^**
	7	−2.21 ± 0.1310 ^Aa^**	−2.07 ± 0.0751 ^Aa^**
	14	−2.22 ± 0.1190 ^Aa^**	−2.08 ± 0.0836 ^Aa^**
	21	−2.84 ± 0.2100 ^Bb^**	−2.08 ± 0.1010 ^Aa^**
Syneresis (%)	1	18.30 ± 2.390 ^Ba^*	12.50 ± 1.460 ^ABb^*
	7	18.43 ± 2.050 ^Ba^*	11.58 ± 1.320 ^Bb^*
	14	22.77 ± 2.970 ^Aa^*	12.23 ± 1.090 ^ABb^*
	21	19.16 ± 2.040 ^Ba^*	14.66 ± 1.600 ^Ab^*
Cohesiveness (N)	1	0.68 ± 0.0456 ^Ba^*	0.68 ± 0.0727 ^Aa^*
	7	0.72 ± 0.039 ^Aa^*	0.68 ± 0.0746 ^Ab^*
	14	0.72 ± 0.0452 ^Aa^*	0.70 ± 0.0739 ^Aa^*
	21	0.73 ± 0.0347 ^Aa^*	0.67 ± 0.0721 ^Ab^*

^1^ Data are the means ± SE (n = 9) of all non-microfluidized goat milk yogurt samples (YC, Y1, Y2) and *microfluidized* (MFYC, MFY1, MFY2) goat milk yogurt samples for the parameters in storage days of 1, 7, 14 and 21, different capital letters in the same column indicate significant difference (* *p* < 0.05, ** *p* < 0.01), different lowercase letters in the same row indicate significant difference (* *p* < 0.05, ** *p* < 0.01).

**Table 9:** The individual effect of storage time on b* value, the viability of yogurt bacteria, and some texture characteristics of goat milk yogurt samples.

Parameter	Storage Time (Day)	$\bar{X}$ ± *S_x_* ^1^
b*	1	7.96 ± 0.3960 ^B^*
	7	8.75 ± 0.1180 ^A^*
	14	8.80 ± 0.1280 ^A^*
	21	8.70 ± 0.1030 ^A^*
Firmness (N)	1	1.43 ± 0.147 ^B^**
	7	1.50 ± 0.151 ^AB^**
	14	1.51 ± 0.161 ^A^**
	21	1.53 ± 0.159 ^A^**
Consistency (N×s)	1	14.45 ± 1.68 ^B^*
	7	14.96 ± 1.72 ^A^*
	14	15.12 ± 1.73 ^A^*
	21	15.15 ± 1.76 ^A^*
*S. thermophilus* (log CFU/g)	1	9.93 ± 0.209 ^B^**
	7	10.98 ± 0.369 ^A^**
	14	10.23 ± 0.276 ^B^**
	21	10.13 ± 0.293 ^B^**
*Lb. delbrueckii* subsp. *bulgaricus* (log CFU/g)	1	8.62 ± 0.183 ^A^**
	7	8.08 ± 0.127 ^B^**
	14	7.36 ± 0.142 ^C^**
	21	6.85 ± 0.217 ^D^**

^1^ Data are the means ± SE (n = 18) of both non-microfluidized and microfluidized goat milk yogurt samples for the parameters in storage days of 1, 7, 14, and 21, different capital letters in the same column indicate a significant difference (* *p* < 0.05, ** *p* < 0.01).

## Data Availability

All data are contained within the article.
